# Genomic and pathogenic characterization of a highly pathogenic chicken infectious anemia virus strain in China

**DOI:** 10.3389/fvets.2026.1893535

**Published:** 2026-07-21

**Authors:** Hongchun Yang, Liuyang Yuan, Hao Chen, Yue Liu, Aobo Xu, Ziqi Ling, Xiaoran Xuan, Shuangshuang Ma, Shufeng Feng, Yaping Wang, Hongbin Si, Gonghe Li, Yang Li, Anding Zhang, Changbo Ou

**Affiliations:** 1 College of Animal Science and Technology, Guangxi University, Nanning, China; 2 Guangxi Yuanfeng Group Pastoral Industry Co. Ltd, Lingshan, China; 3 China Animal Health and Epidemiology Center, Qingdao, Shandong, China; 4 College of Veterinary Medicine, Huazhong Agricultural University, Wuhan, China; 5 Guangxi Key Laboratory of Animal Reproduction, Breeding and Disease Control, Nanning, China; 6 Guangxi Zhuang Autonomous Region Engineering Research Center of Veterinary Biologics, Nanning, China

**Keywords:** chicken infectious anemia virus, genomic characterization, immunosuppression, pathogenicity, virulence

## Abstract

Chicken infectious anemia virus (CIAV) is a major immunosuppressive pathogen of poultry, causing aplastic anaemia, lymphoid atrophy, and severe haematopoietic dysfunction in young chicks, thereby posing a substantial threat to global poultry health and production. In this study, a novel highly pathogenic CIAV strain, designated CIAV-GDHY230813, was isolated from young Mahuang chickens in China and subjected to comprehensive molecular and pathogenic characterization. Whole-genome sequencing and phylogenetic analysis revealed that CIAV-GDHY230813 belongs to I-a branch. Notably, the VP1 protein harbored a glutamine residue at position 394, a molecular marker strongly associated with high virulence, together with multiple amino acid substitutions, insertions, and deletions across the coding regions. Pathogenicity experiments in specific-pathogen-free (SPF) chicks demonstrated that infection with CIAV-GDHY230813 resulted in pronounced growth retardation, severe damage to immune organs, and increased mortality. From 3 to 21 days post-infection (dpi), body weights of infected chicks were significantly lower than those of the control group (*p* < 0.01). Marked thymic and bursal atrophy, splenomegaly, and significantly reduced haematocrit levels were observed, indicating severe anaemia. Furthermore, CIAV infection led to a marked reduction in antibody titres against Newcastle disease virus vaccination by 4-fold to 8-fold, reflecting substantial suppression of humoral immune responses. Quantitative analysis of viral distribution showed significantly elevated viral loads in blood, liver, thymus, spleen, and bursa of Fabricius at both 14 and 21 dpi (*p* < 0.001), with peak levels detected at 21 dpi. Collectively, these findings demonstrate the strong replicative capacity and high pathogenic potential of CIAV-GDHY230813, providing valuable insights into the molecular epidemiology and pathogenic mechanisms of CIAV in China, supporting improved surveillance and control strategies, and laying a solid foundation for the development of effective vaccines against CIAV.

## 1 Introduction

Chicken infectious anemia (CIA), caused by CIAV, is a highly contagious immunosuppressive disease that poses a substantial threat to the global poultry industry. The virus primarily induces severe immunosuppression in young chicks, characterized by aplastic anemia, systemic lymphoid atrophy, and hematopoietic dysfunction (1, 2). In adult chickens, CIAV infection is generally subclinical; however, it markedly compromises production performance and induces persistent immunosuppression, thereby reducing vaccine efficacy and increasing susceptibility to secondary infections by a wide range of pathogenic microorganisms, thereby threatening the economic sustainability of poultry operations (3). Consequently, CIAV represents a major challenge to the sustainability and economic viability of modern poultry production systems. CIAV was first isolated in Japan in 1979, and efficient *in vitro* propagation was subsequently achieved using MDCC-MSB1 cells (4). Since then, the virus has disseminated worldwide and becomes endemic in nearly all major poultry-producing regions, emerging as one of the most important immunosuppressive pathogens affecting poultry.

CIAV can be transmitted both vertically and horizontally. Vertical transmission from infected breeder hens to their offspring results in early infection and severe disease in chicks, whereas horizontal transmission via feces, secretions, and other excreta facilitates rapid spread within flocks (5, 6). In China, CIAV was first reported in Heilongjiang Province in 1992 and has since spread extensively, becoming prevalent in most poultry-producing provinces nationwide (7). Recent serological surveys indicate persistently high CIAV seroprevalence rates in commercial chicken farms across Jiangsu, Zhejiang, Anhui, Guangxi, and other regions, exceeding 70% in some areas, which confirms the stable endemic status of CIAV in Chinese poultry populations (8). Notably, CIAV frequently co-infects chickens together with other immunosuppressive viruses, including Marek’s disease virus (MDV), reticuloendotheliosis virus (REV), subgroup J avian leukosis virus (ALV-J), and fowl adenovirus (FAdV). Such co-infections exacerbate clinical manifestations, increase morbidity and mortality, severely impair host immune function, and substantially reduce the protective efficacy of routine vaccinations, thereby resulting in significant economic losses to the poultry industry (9–11).

According to the International Committee on Taxonomy of Viruses (ICTV), CIAV is classified within the genus Gyrovirus of the family Anelloviridae (12, 13). It is a small, non-enveloped, single-stranded circular DNA virus with icosahedral virions approximately 23.5 nm in diameter (14). The CIAV genome is typically 2,298 bp in length, although some strains extend to 2,319 bp due to the presence of an additional 21-bp direct repeat (DR). The number of DRs is closely associated with viral adaptability to host cells (15, 16). The viral genome contains three partially overlapping open reading frames (ORFs) encoding VP1 (1,350 bp, 51.6 kDa), VP2 (651 bp, 24.0 kDa), and VP3 (366 bp, 13.6 kDa) (17). VP1, the sole structural protein, is essential for virion assembly and harbors major antigenic determinants, forming neutralizing epitopes together with VP2. VP2 functions as a scaffold protein required for proper VP1 folding and virion assembly and also exhibits protein phosphatase activity critical for viral replication. VP3, also known as apoptin, selectively induces apoptosis in transformed and tumor cells and plays an important role in CIAV-induced immunosuppression, hemorrhage, and anemia (18–20). The VP2 and VP3 coding regions are fully overlapping, while the VP1 coding region partially overlaps with VP2. A 300-bp non-coding region (NCR) upstream of VP2 contains promoters, transcriptional regulatory elements, polyadenylation signals, and transcription factor binding sites, which play a pivotal role in regulating viral transcription (21).

Despite significant advances in CIAV surveillance, molecular characterization, and epidemiological studies in China, effective control of CIAV remains challenging. Currently, no commercially available CIAV vaccine has been approved for use in China, and prevention strategies rely mainly on biosecurity measures and routine pathogen monitoring. However, these approaches have proven insufficient to effectively curb viral transmission under intensive production conditions (22). Although single CIAV infection typically results in relatively low mortality, the severe immunosuppression induced by the virus, together with frequent co-infections and reduced vaccine efficacy against Marek’s disease and infectious bursal disease, leads to substantial economic losses (23). Therefore, systematic isolation of circulating CIAV strains and comprehensive investigations into their genomic characteristics, evolutionary dynamics, and pathogenic diversity are essential for enriching molecular epidemiological data, facilitating viral tracing and evolutionary studies, and providing a scientific basis for the development of targeted control strategies, novel vaccines, and optimized diagnostic tools. In this study, we isolated and characterized a CIAV strain from commercial chickens in South China and conducted detailed analyses of its genomic features, evolutionary relationships, and pathogenicity. Our findings aim to elucidate the molecular epidemiology and pathogenic mechanisms of CIAV circulating in this region, thereby providing theoretical and technical support for effective disease control and prevention strategies in the Chinese poultry industry.

## 2 Materials and methods

### 2.1 Clinical samples

In August 2023, young Mahuang chickens at a farm in Heyuan City of Guangdong Province, China, developed symptoms of anemia, which was suspected of being infected with CIAV.

### 2.2 CIAV detection

Tissue samples were collected from diseased chickens, including the bursa of Fabricius and spleen. The tissues were homogenised and subjected to three freeze–thaw cycles at −80 °C, followed by centrifugation at 5,000 rpm for 10 min to obtain the supernatants. Viral genomic DNA was extracted using the FastPure Viral DNA/RNA Mini Kit (Vazyme, Nanjing, China), in accordance with the manufacturer’s instructions. Specific primers were designed based on the genomic sequence of the CIAV reference strain Cuxhaven-1 (GenBank accession number M55918.1) using Primer-BLAST, as follows: forward primer: 5′- AATGAACGCTCTCCAAGAAG-3′; reverse primer: 5′- AGCGGATAGTCATAGTAGAT -3′. PCR amplification was performed using 2 × Rapid Taq Master Mix (Vazyme, Nanjing, China). The expected amplicon size was 582 bp. The PCR cycling conditions were as follows: initial denaturation at 95 °C for 5 min; 35 cycles of denaturation at 95 °C for 30 s, annealing at 55 °C for 15 s, and extension at 72 °C for 15 s; followed by a final extension at 72 °C for 5 min. Finally, 5 μL of each PCR product was analysed by electrophoresis on a 1% agarose gel and visualized under ultraviolet illumination.

### 2.3 Virus isolation

The supernatant derived from PCR-positive tissues was filtered through a 0.22 μm sterile membrane filter and subsequently treated with penicillin–streptomycin (2,000 IU/mL; Solarbio, Beijing, China) at 4 °C for approximately 1 h. Thereafter, 200 μL of a ten-fold dilution was inoculated into the yolk sac of 7-day-old SPF chicken embryos. The embryos were incubated at 37 °C, and those that died within 24 h post-inoculation were excluded from further analysis. At 16 days of embryonation, the liver, spleen, and decapitated and de-limbed embryo bodies were aseptically collected, homogenised in sterile PBS, and subjected to three freeze–thaw cycles at −80 °C. Following centrifugation at 5,000 rpm for 5 min, the supernatants were harvested and subjected to three consecutive blind passages for virus isolation.

### 2.4 Viral genome sequencing

Viral genomic DNA was extracted using the FastPure Viral DNA/RNA Mini Kit (Vazyme, Nanjing, China), in accordance with the manufacturer’s instructions. The full-length CIAV genome was amplified using three pairs of primers (Table 1), designed based on the reference strain Cuxhaven-1 (GenBank accession no. M55918.1) with Primer-BLAST. The purified PCR products were ligated into the pMD18-T vector (TaKaRa, Beijing, China) and subjected to commercial sequencing (Sangon Biotech, Shanghai, China). Sequence assembly and analysis were performed using SeqMan software (Lasergene v7.1, DNASTAR, Madison, WI, USA) to obtain the complete CIAV genome.

**Table 1:** Primers for PCR detection of CIAV VP1, VP2, and VP3.

Name	Sequence (5′-3)	Length (bp)
CIAV-F-1	ATTCCGAGTGGTTACTATTCC	861
CIAV-R-1	CGTCTTGCCATCTTACAGTCTTAT	
CIAV-F-2	CGAGTACAGGGTAAGCGAGCTAAA	990
CIAV-R-2	TGCTATTCATGCAGCGGACTT	
CIAV-F-3	ACGAGCAACAGTACCCTGCTAT	806
CIAV-R-3	CTGTACGTGCTCCACTCGTT	

### 2.5 Homology and phylogenetic analysis

The assembled CIAV gene sequences were processed using SeqMan (Lasergene 7.1). Based on whole-genome CIAV sequences available in GenBank, 49 reference strains from different periods and regions were selected (Supplementary Table S1). Nucleotide sequence similarity between the isolate and reference strains was analyzed with MEGA 7.0. Phylogenetic trees were constructed from whole-genome sequences. Sequence alignment of whole genomes and specific genes was performed using the Clustal W algorithm in MEGA 7.0, and trees were generated via iqtree3.1.1 using maximum likelihood, the optimal nucleotide substitution model was automatically selected as TN + F + R2 with 1,000 bootstrap replicates. Amino acid sequences of the isolate and reference strains were compared to identify mutations in key functional regions.

### 2.6 Pathogenicity of the CIAV isolate to SPF chicks

Fifty-nine one-day-old SPF chicks were randomly allocated into four experimental groups and one control group. Group A served as the negative control (*n* = 20) and received an intramuscular injection of 0.2 mL sterile 0.9% saline into the leg muscle at one day of age. Group B constituted the CIAV-infected group (*n* = 15) and was inoculated intramuscularly in the leg with 0.2 mL of the CIAV-GDHY230813 isolate at one day of age. Group C was the NDV LaSota vaccine-immunised group (*n* = 12), which received 0.2 mL of the LaSota vaccine via the intranasal and intraocular routes at seven days of age. Group D was the CIAV and NDV LaSota co-infection group (*n* = 12); chicks were inoculated intramuscularly with 0.2 mL of CIAV-GDHY230813 at one day of age and subsequently immunised with the LaSota vaccine (0.2 mL per bird) via the intranasal and intraocular routes at seven days of age. All experimental birds were housed under independent isolation conditions. Groups A and B were maintained until 21 days of age, while Groups C and D were reared until 28 days of age. Birds were provided with ad libitum access to feed and water throughout the experimental period. Clinical signs were monitored daily, and mortality was recorded.

At 7, 14, and 21 days of age, five birds were randomly selected from each of groups A and B. From each bird, 2 mL of anticoagulated blood and 2 mL of non-anticoagulated blood were collected via the wing vein. White blood cell counts, red blood cell counts, and haematocrit values were determined using an automated haematology analyser. Pharyngeal and cloacal swabs were collected for the detection of viral shedding. Tissue samples from the thymus, trachea, spleen, liver, kidney, proventriculus, femoral bone marrow, bursa of Fabricius, and caecal tonsils were collected for histopathological examination and viral load quantification. In addition, immune organs (thymus, bursa of Fabricius, and spleen) were harvested from another five randomly selected chicks and weighed. The immune organ index was calculated as immune organ weight (mg) divided by body weight (g) to evaluate the impact of CIAV infection on primary immune organs.

To evaluate the effect of CIAV infection on antibody responses following routine vaccination, at 14, 21, and 28 days of age, three chicks were randomly selected from groups C and D, and non-anticoagulated blood was collected from the jugular vein to separate serum. Serum antibody levels were assessed through both a conventional hemagglutination inhibition (HI) assay.

To further assess growth performance and survival, an additional 30 one-day-old SPF chicks were randomly assigned to two equal groups. The control group received an intramuscular injection of 0.2 mL sterile saline into the leg at one day of age, whereas the challenged group was inoculated intramuscularly with 0.2 mL of the CIAV-GDHY230813 isolate. All birds were reared in isolation until 21 days of age with unrestricted access to feed and water. Clinical signs, body weight, and mortality were recorded daily. Growth curves were generated based on body weight measurements, deceased chicks were subjected to immediate necropsy, and survival rates were calculated for each group. All animal experiments were performed in strict compliance with the institution’s guidelines on animal welfare.

### 2.7 Histopathological examination

The collected thymus, spleen, bursa of Fabricius and liver were fixed in 10% formalin and then embedded in paraffin, sectioned, stained with hematoxylin and eosin (H&E), and observed using a light microscope.

### 2.8 Viral load determination in tissues

Based on the Diagnostic Techniques for Chicken infectious anemia virus (Industry Standard, NY/T 1187–2019), the viral load of each organ was detected by SYBR Green I Real-time PCR to analyze the replication of the CIAV-GDHY230813 strain. The specific primers (forward primer: 5’-GCAGGGGCAAGTAATTTCAA-3′; reverse primer: 5’-GCCACACAGCGATAGAGTGA-3′) were synthesized by Sangon Biological Technology (Shanghai, China). Total DNA was extracted from anticoagulated blood and from the thymus, spleen, liver, and bursa of Fabricius tissues collected from Groups A and B at 7, 14, and 21 days of age were extracted using the TIANamp Genomic DNA Kit (TIANGEN, Beijing, China). Amplification was performed using ChamQ Universal SYBR qPCR Master Mix (Vazyme, Nanjing, China) on a TGreat Real 96 Real-Time PCR System (TIANGEN) under the following conditions: incubation at 95 °C for 30 s; 35 cycles of 95 °C for 10 s and 60 °C for 30 s; the instrument’s default melting curve program was applied for data collection.

### 2.9 Statistical analysis

GraphPad Prism (version 8.3.0) and SPSS (version 27.0) software were used for statistical analysis, and the following symbols were used to indicate significant differences between groups: *p < 0.05, **p < 0.01, ***p < 0.001, ns, not significant.

## 3 Results

### 3.1 Isolation and identification of CIAV

**Figure 1:**
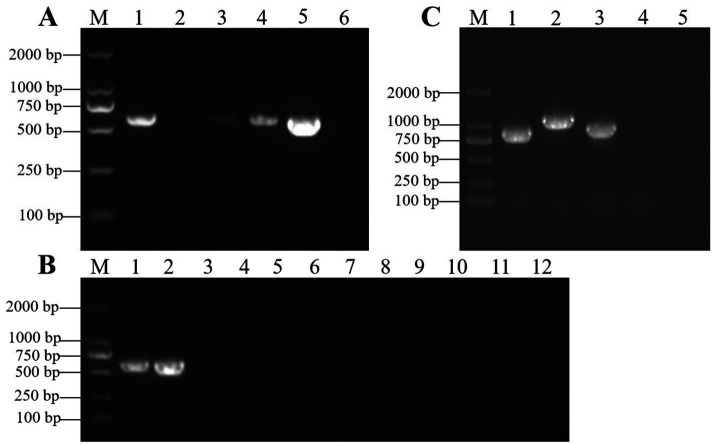
PCR detection for CIAV. The genomic DNA extracted from the clinical samples were detected by PCR using specific primers of CIAV **(A)** PCR detection results of clinical samples. M: DNA Marker DL2000, 1–4: Samples, 5: Positive Control, 6: Negative Control. **(B)** Detection Results of Exogenous Viruses in Poultry by PCR. 1: CIAV, 2: CIAV Positive Control, 3: CIAV Negative Control, 4–12: NDV, IBV, ALV, FADV, ARV, MDV, ILTV, AIV, IBDV. **(C)** Whole genome amplification of CIAV-GDHY230813 isolate. M: DNA Marker DL2000, 1–3: Samples, 4–5: Negative Control.

The CIAV-positive tissue homogenate supernatant was inoculated into the yolk sac of 7-day-old SPF chicken embryos and incubated for 9 days. After three blind passages, viral DNA was extracted from homogenized liver, spleen, and embryo tissues for PCR detection (Figure 1A). The amplified target fragments were subsequently cloned into the pMD18-T vector and transformed into *Escherichia coli* DH5α competent cells. Positive clones were screened by PCR, and recombinant plasmids were purified and sequenced by Sangon Biotech (Shanghai, China). BLAST analysis confirmed the isolate as CIAV, and the strain was designated CIAV-GDHY230813.

The viral genome was subjected to PCR identification, and a CIAV-specific band was detected at the position of 582 bp. The detection results for common poultry viruses such as NDV, IBV, ALV, FADV, ARV, MDV, ILTV, AIV, and IBDV showed that, except for CIAV, no other common poultry pathogens were detected (Figure 1B).

To determine the complete genome sequence, three pairs of specific primers were used to amplify overlapping fragments covering the entire viral genome. Agarose gel electrophoresis confirmed successful amplification of three fragments with expected sizes of 861 bp, 990 bp, and 806 bp, respectively (Figure 1C). These PCR products were gel-purified, cloned individually into the pMD18-T vector, and sequenced. The resulting sequences were assembled using SeqMan software, yielding a complete genome of 2,298 bp.

### 3.2 CIAV-GDHY230813 belongs to the I-a branch

**Figure 2:**
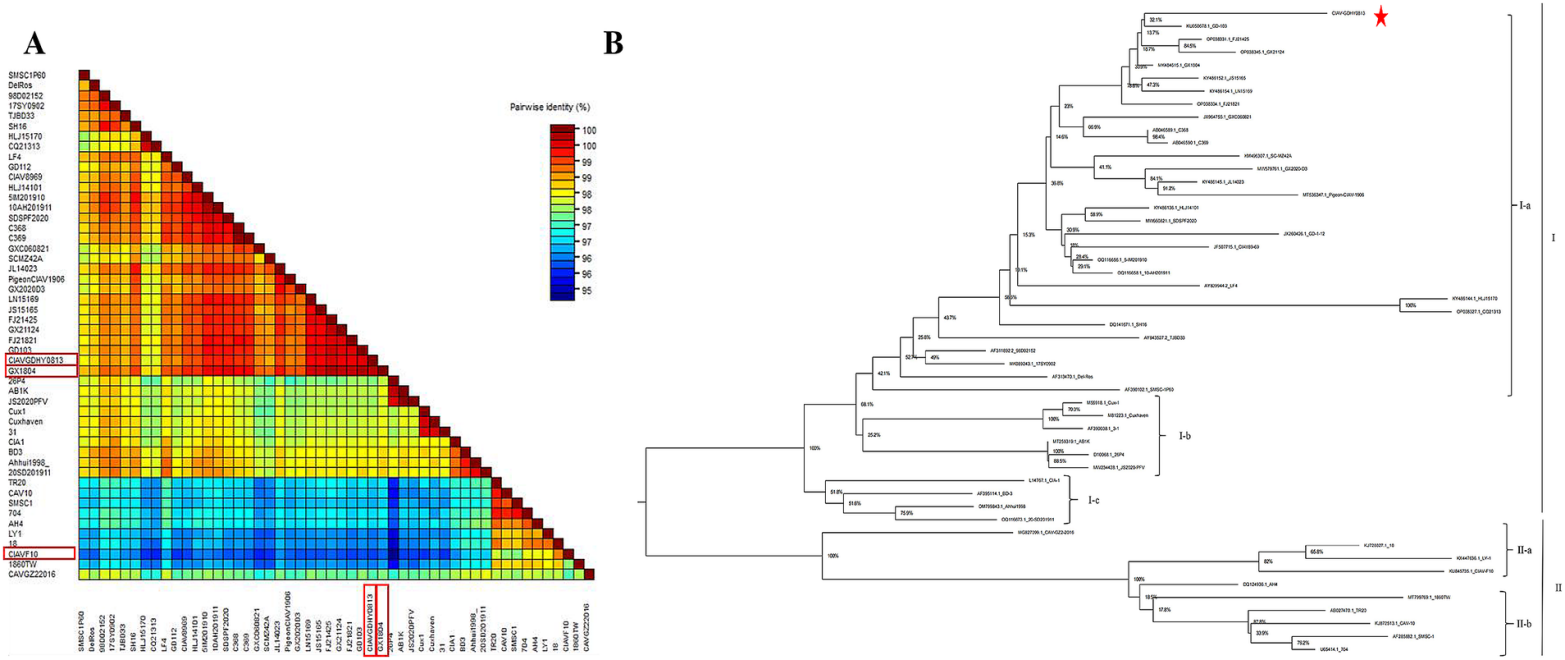
Homology comparison and phylogenetic analysis of the CIAV isolate. **(A)** Homology analysis was performed on the complete genome sequence of the CIAV-GDHY230813 isolate and 49 reference strains (including both domestic and international strains) retrieved from the GenBank database using the MegAlign program in the Lasergene 7.1 software package. **(B)** Phylogenetic tree based on the complete genome. Phylogenetic trees were constructed from whole-genome sequences. Sequence alignment of whole genomes and specific genes was performed using the Clustal W algorithm in MEGA 7.0, and trees were generated via iqtree3.1.1 using maximum likelihood, the optimal nucleotide substitution model was automatically selected as TN + F + R2 with 1,000 bootstrap replicates.

The complete genome sequence of CIAV-GDHY230813 was aligned with those of 49 reference CIAV strains retrieved from GenBank using MegAlign software (Figure 2A). Nucleotide sequence identity ranged from 96.0 to 99.7%. The highest homology (99.7%) was observed with strain GX1804 (MK484615.1), whereas the lowest identity (96.0%) was shared with strain CIAV-F10 (KU845735.1).

Using the maximum likelihood (ML) method with iqtree 3.1.1, the optimal nucleotide substitution model was automatically selected as TN + F + R2. Based on the topology of the phylogenetic tree constructed from 50 reference sequences, the virus was divided into two major groups (I and II): Group I further subdivided into subgroups I-a, I-b, and I-c, while Group II was further divided into subgroups II-a and II-b. CIAV-GDHY230813 belongs to the I-a branch and exhibits close genetic relationships with the GD-103 and FJ21425 strains, whereas it diverges significantly from vaccine strains such as Cux-1 and 26P4, indicating a distant phylogenetic relationship (Figure 2B). These findings suggest that CIAV-GDHY230813 likely represents a wild-type strain circulating in the wild.

### 3.3 CIAV-GDHY230813 has highly pathogenic sequence characteristics

VP1 is a structural protein with relatively low conservation in the CIAV genome, and it directly influences viral replication rate and cellular infectivity. Sequence alignment revealed that the hypervariable region (amino acids 139–157) of VP1 in CIAV-GDHY230813 was completely conserved compared with reference strains (Supplementary Figure S1). Residues at positions 139 and 144 were lysine (K) and glutamic acid (E), respectively, which are associated with high infectivity in MDCC-MSB1 cells. Importantly, residue 394 was glutamine (Q), a molecular marker strongly correlated with high virulence, suggesting that CIAV-GDHY230813 is highly pathogenic. In addition, amino acids at positions 75, 89, 125, and 141 were valine (V), threonine (T), leucine (L), and glutamine (Q), respectively, consistent with the molecular profile of highly virulent strains (Table 2). Two additional amino acid substitutions, D192G and L376I, were also noted. Since they lie outside the classical hypervariable region, they were consequently omitted from the Table.

**Table 2:** The known virulence determining sites in VP1 of the isolates.

Isolates/amino acidsites	75	89	125	139	141	144	157	287	394
Majority virulent strains	V	T	I	Q	Q	Q	V	T	Q
Low virulent strains	I	A	L	K	E	E	M	S	H
CIAV-GDHY230813	V	T	L	K	Q	E	V	S	Q

VP2, a nonstructural protein essential for viral replication and assembly, displayed a high degree of sequence conservation. Only a single amino acid substitution (G24E) was observed in CIAV-GDHY230813, while residues at positions 95 and 97 remained cysteine (C), indicating preservation of key functional domains (Supplementary Figure S2). VP3, also known as apoptin, contributes to viral pathogenicity by inducing apoptosis in host cells. No amino acid mutations were detected in VP3 of CIAV-GDHY230813 (Supplementary Figure S3), suggesting that its structure and function are likely intact and may support efficient viral replication and pathogenicity.

Collectively, these sequence features, particularly those at established virulence-determining sites, indicate that CIAV-GDHY230813 possesses a molecular profile characteristic of highly pathogenic strains.

### 3.4 The CIAV-GDHY230813 strain was highly pathogenic to SPF chickens

**Figure 3:**
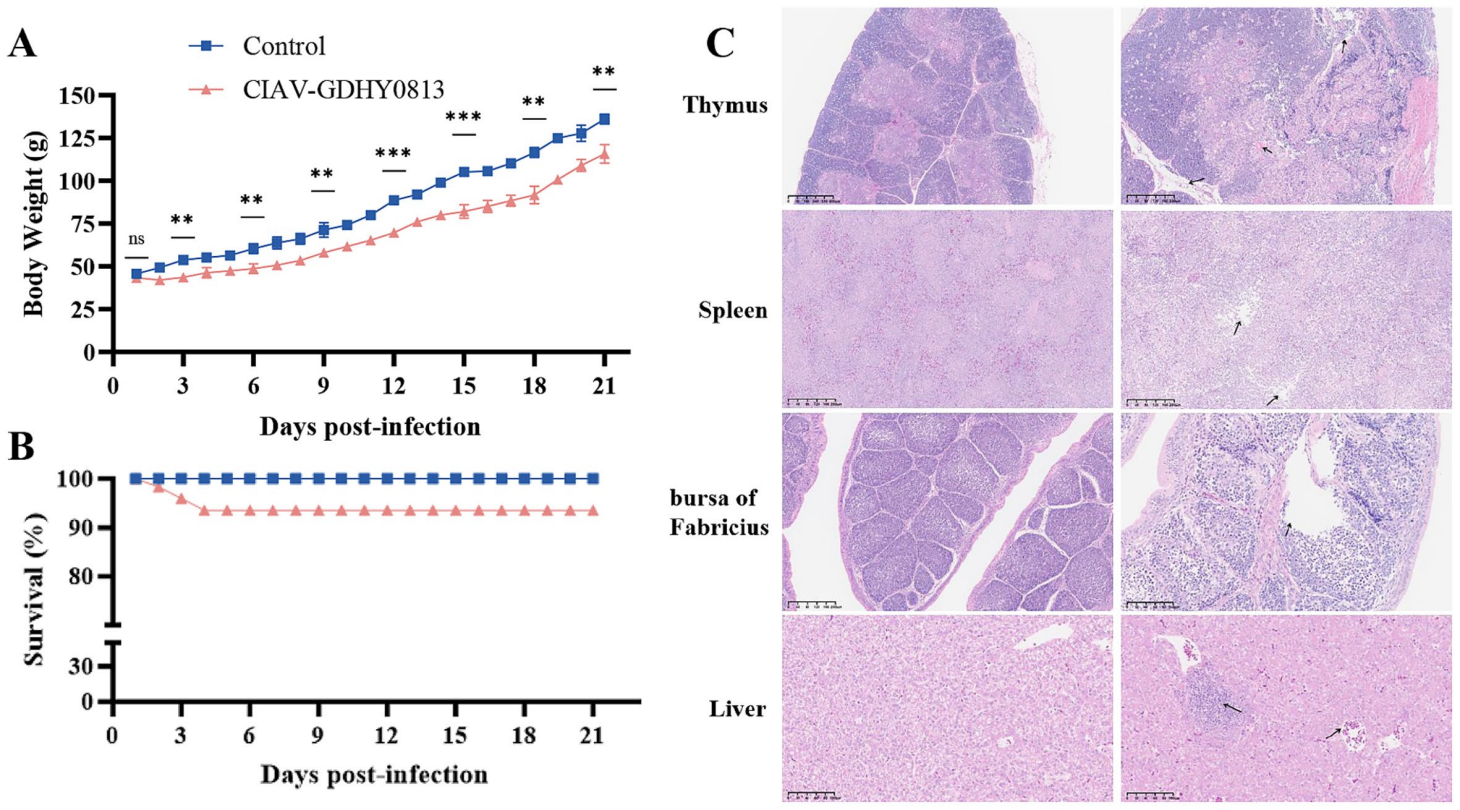
Pathogenicity analysis of CIAV-GDHY230813 on SPF chickens. **(A)** Changes in body weight of SPF chickens. **(B)** Survival rate of SPF chickens. **(C)** Histopathological lesions change in the thymus, spleen, bursa of Fabricius and liver the control group and the CIAV-GDHY230813 group on d 7 postinfection. Lesions are marked with a black arrow in the CIAV-GDHY230813 group (Thymus ×100, Spleen ×100, bursa of Fabricius × 200, Liver × 200).

Following intramuscular inoculation of 1-day-old SPF chicks with CIAV-GDHY230813, the challenged group gradually exhibited clinical signs including lethargy, ruffled feathers, reduced feather luster, and growth retardation, whereas control chicks remained clinically normal throughout the observation period. Body weights in the challenged group were consistently lower than those in the control group and were significantly reduced at 3, 6, 9, 12, 15, 18, and 21 days of age (*p* < 0.01). Moreover, the magnitude of weight difference increased progressively over time, indicating that CIAV-GDHY230813 infection severely impaired chick growth and development (Figure 3A). No mortality occurred in the control group, yielding a survival rate of 100%. In contrast, mortality in the challenged group was primarily observed within the first 5 dpi, approximately 6.7% mortality in SPF chicks (Figure 3B).

Histopathological examination revealed marked pathological alterations in the thymus, spleen, bursa of Fabricius, and liver of infected chicks. The thymus exhibited capsule shrinkage, reduced cortical and medullary lymphocyte density, indistinct corticomedullary boundaries, widened interlobular septa, and connective tissue hyperplasia. The spleen showed disruption of normal architecture, blurring of red and white pulp boundaries, expansion of red pulp, extensive lymphocyte necrosis and depletion, and reticular cell proliferation. The bursa of Fabricius displayed severe lymphoid depletion, follicular atrophy, widened interfollicular spaces, and reticular cell hyperplasia. Hepatic lesions included hepatocellular degeneration, interstitial hemorrhage, and infiltration of lymphocytes and monocytes. No remarkable pathological changes were observed in tissues from the control group (Figure 3C).

### 3.5 CIAV-GDHY230813 induces severe Anemia in infected chickens

**Figure 4:**
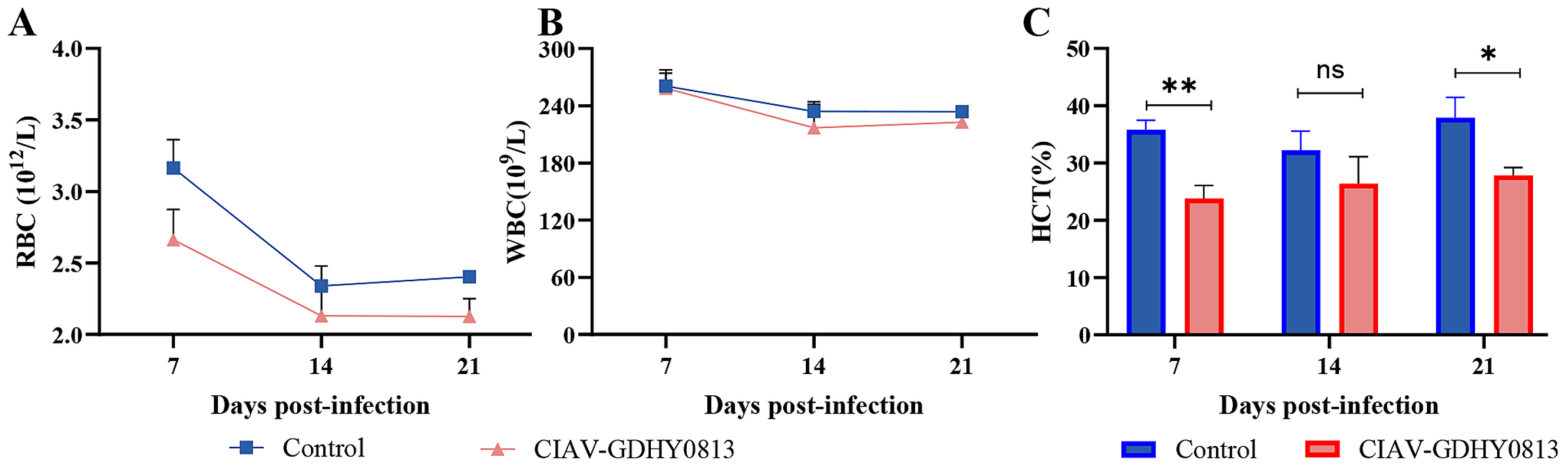
Effects of CIAV-GDHY230813 on White Blood Cells, Red Blood Cells and Hematocrit in SPF Chickens. **(A)** Red blood cell numbers of chickens in different groups. **(B)** White blood cell numbers of chickens in different groups. **(C)** Hematocrit of chickens in different groups.

Routine hematological analyses were conducted to evaluate the impact of CIAV-GDHY230813 on hematopoietic function. Compared with controls, infected chicks exhibited significantly reduced red blood cell (RBC) and white blood cell (WBC) counts (Figures 4A,B). Hematocrit (HCT) values in the control group remained above 30% throughout the experimental period. In contrast, HCT levels in the challenged group were significantly decreased at 7 and 21 days of age (*p* < 0.05). Although the difference at 14 days did not reach statistical significance, HCT values in infected chicks remained consistently lower than those of controls. The most pronounced anemia was observed at 7 dpi, followed by partial recovery at later time points; however, HCT levels remained below 30% throughout the study (Figure 4C).

### 3.6 CIAV-GDHY230813 causes atrophy of immune organs and suppresses immune responses in chickens

**Figure 5:**
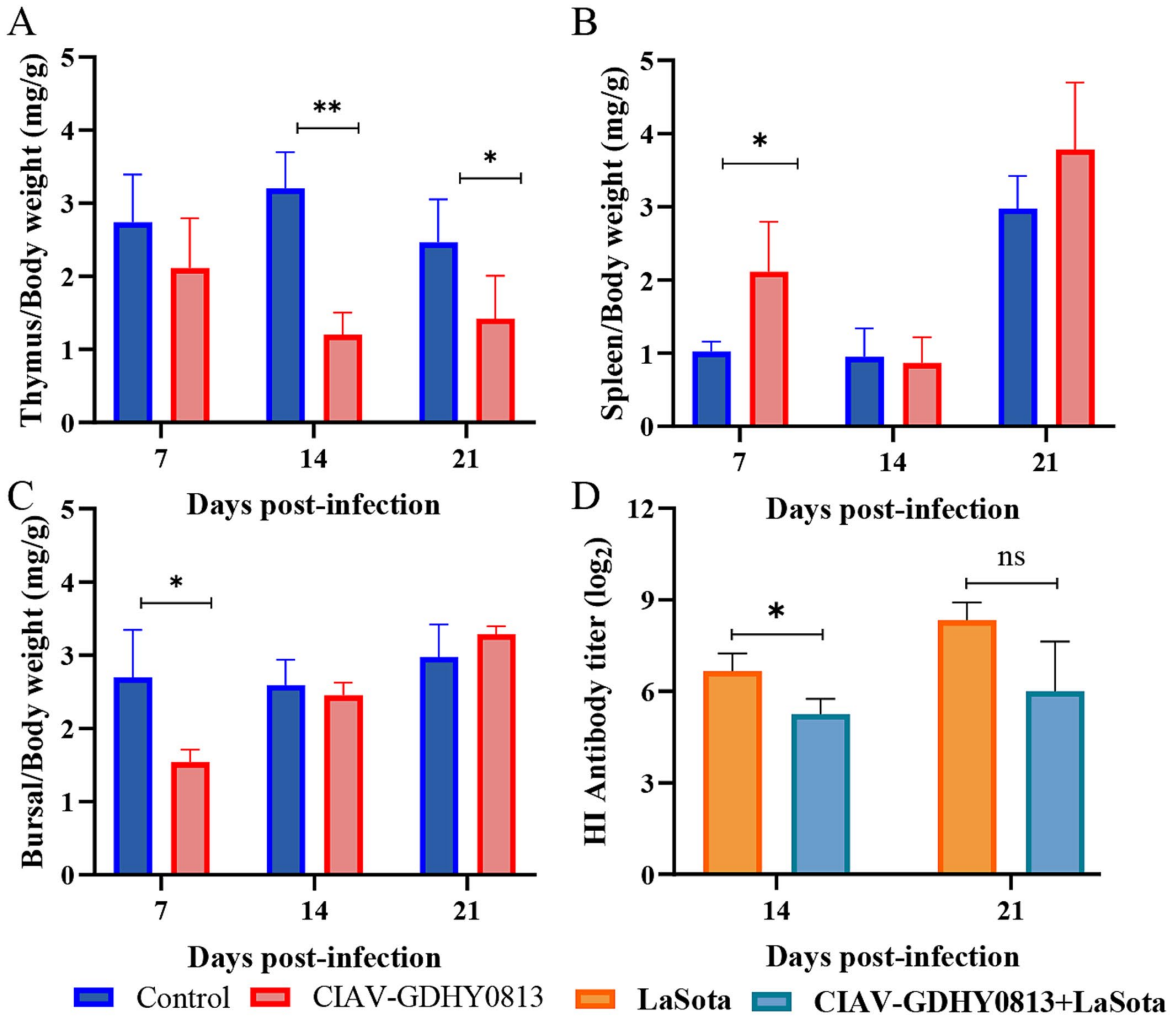
Effect of CIAV-GDHY230813 on the Immune Organ Index and Immune Response of SPF Chickens. **(A)** Thymus index. **(B)** Spleen index. **(C)** bursal index. **(D)** Effect of CIAV-GDHY230813 infection on antibody levels after NDV vaccine. The *y*-axis represents log2-transformed antibody titres, *indicates significant difference between CIAV-infected/vaccinated group and vaccinated-only group, *p* < 0.05.

To assess the effects of CIAV-GDHY230813 on immune organ development, thymus, spleen, and bursa of Fabricius indices were determined in randomly selected chicks from each group at 7, 14, and 21 dpi. The thymus index in the challenged group was significantly reduced compared with that in the control group (*p* < 0.05), indicating marked suppression of thymic development (Figure 5A). The spleen index was generally higher in infected chicks, reflecting splenic enlargement, although a slight decrease was observed at 14 dpi, which did not reach statistical significance (Figure 5B). Bursa index was significantly lower in the challenged group at 7 dpi (*p* < 0.05), suggesting early inhibition of bursal development, whereas no significant differences were detected at 14 or 21 days (Figure 5C).

To evaluate the immunosuppressive effects of CIAV-GDHY230813 on vaccine-induced immunity, serum samples were collected at 14 and 21 days following immunization with Newcastle disease virus (NDV) vaccine, and antibody titers were measured using the hemagglutination inhibition (HI) assay. At 14 days of age, NDV-specific antibody titers in the infected group were significantly lower than those in the non-infected immunized group (*p* < 0.05), indicating a pronounced inhibitory effect of CIAV on humoral immune responses. Although antibody levels increased at 21 days of age, titers in the challenged group remained significantly lower than those of the control group, demonstrating sustained immunosuppression induced by CIAV infection (Figure 5D).

### 3.7 Virus shedding status and viral load in different tissues of SPF chickens

Pharyngeal and cloacal swabs were collected from infected and control chicks at 7, 14, and 21 days of age and analyzed by PCR to assess viral shedding (Table 3). All samples from the control group remained negative throughout the experiment. In the challenged group, viral detection rates were low at 7 days, with only 25% positivity in both pharyngeal and cloacal swabs. By 14 days, detection rates increased to 75% in pharyngeal swabs and 100% in cloacal swabs. At 21 days, both sample types exhibited 100% positivity, indicating active and persistent viral shedding.

**Table 3:** The virus shedding of experimental groups after CIAV-GDHY230813 strain infection.

Groups	Throat			Cloaca		
	7 dpi	14 dpi	21 dpi	7 dpi	14 dpi	21 dpi
Control	0/4	0/4	0/4	0/4	0/4	0/4
CIAV-GDHY230813	1/4	3/4	4/4	1/4	4/4	4/4

**Figure 6:**
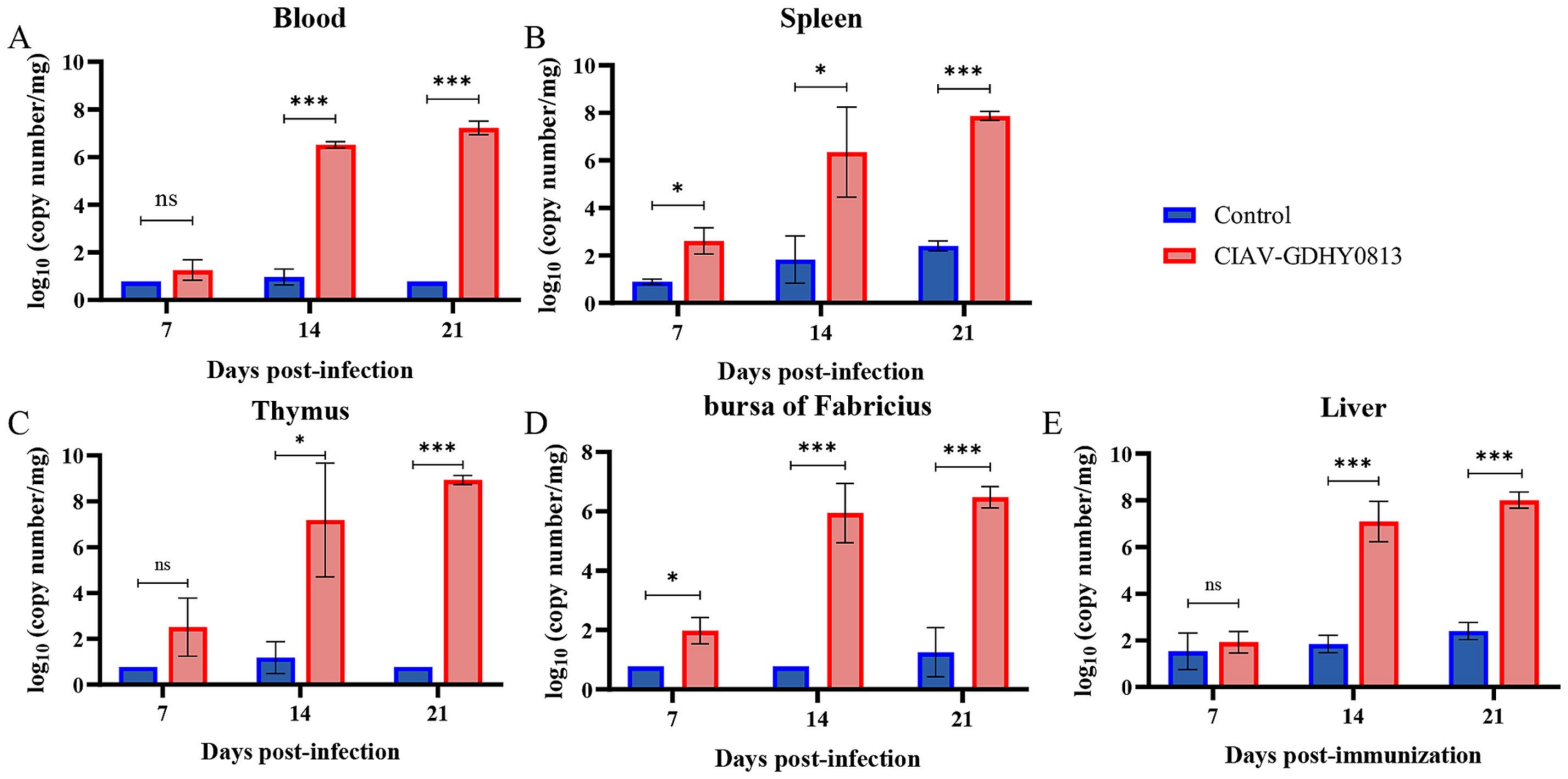
Virus load in different tissues of SPF chickens after CIAV infection. On 7, 14, and 21 days of age of SPF checks, blood **(A)** spleen **(B)** thymus **(C)**, bursa of Fabricius **(D)** and liver **(E)** samples were collected from chicks, after extracting tissue DNA, qPCR was used to detect the CIAV viral load in each tissue.

To further characterize viral distribution, blood, thymus, liver, spleen, and bursa of Fabricius samples were collected at 7, 14, and 21 days post-infection and subjected to qPCR (Figure 6). At 7 days, viral loads were generally low; however, significantly higher copy numbers were detected in the spleen and bursa of infected chicks compared with controls (*p* < 0.05), whereas no significant differences were observed in blood, liver, or thymus (*p* > 0.05). As infection progressed, viral loads increased markedly in all examined tissues. At 14 and 21 days, viral copy numbers in blood and all sampled organs of the challenged group were significantly higher than those of controls (*p* < 0.001), reaching peak levels at 21 days. These findings indicate that CIAV-GDHY230813 is capable of sustained and efficient replication in peripheral blood and major immune organs, demonstrating strong replicative capacity and systemic dissemination.

## 4 Discussion

Recent epidemiological investigations have demonstrated that CIAV infection, either alone or in combination with other pathogens, is widespread in commercial poultry flocks, posing a substantial threat to the sustainable development of the poultry industry in China. In the present study, a CIAV strain, designated CIAV-GDHY230813, was successfully isolated from clinically suspected samples collected in southern China. Following inoculation into the yolk sacs of SPF chicken embryos, the virus was efficiently propagated, and complete genome sequencing revealed a genome length of 2,298 bp.

Homology analysis indicated that CIAV-GDHY230813 shares 96.0–99.7% nucleotide sequence similarity with domestic and international reference strains. It exhibited the highest similarity (99.7%) with strain GX1804 (MK484615.1) and the lowest (96.0%) with strain CIAV-F10 (KU845735.1). Phylogenetic trees based on the complete genome were largely consistent. Among 50 strains classified into two major groups (I and II), CIAV-GDHY230813 fell within Group I-a, showing close genetic relatedness to circulating strains from southern China such as GD-103 (Guangdong) and FJ21425 (Fujian), but distant from foreign vaccine strains including 26P4 and Cux-1. These findings suggest that CIAV-GDHY230813 likely represents a wild-type strain circulating in the wild (8). Although recombination analysis revealed no genetic recombination in CIAV-GDHY230813, previous studies have reported isolates from contaminated attenuated vaccines that are closely related to foreign strains (3). Thus, the potential for increased genetic diversity in CIAV warrants attention, and vigilance against emerging recombinant viruses remains necessary.

The VP1 protein, the sole structural component of CIAV, is also its most variable region. VP1 serves as the major capsid protein and key immunogen, located on the virion surface and subject to host immune selection pressure, which drives high sequence variability (24). The region spanning amino acids 39–157 is considered hypervariable. Substitutions at positions 139 and 144 to glutamine (Q) have been shown to markedly reduce viral replication efficiency and infectivity in MDCC-MSB-1 cells (25). Moreover, position 394 is an important virulence determinant; mutation from glutamine (Q) to histidine (H) significantly attenuates pathogenicity. In CIAV-GDHY230813, residues 139 and 144 were identified as lysine and glutamic acid, respectively, and residue 394 remained glutamine, a molecular profile consistent with highly pathogenic strains recently isolated in Guangdong and Guangxi provinces, furthermore, residues 75, 89, 125, 141 and 144 are identical to those of known highly virulent strains (3). Genetic analysis identified the Q394 mutation on VP1, a well-established molecular marker of highly pathogenic CIAV strains. Combined with obvious clinical symptoms and severe atrophy of immune organs in infected chickens, we preliminarily defined this strain as highly pathogenic. Nevertheless, the mortality rate in this study was only 6.7%, which was lower than the mortality caused by several classic hypervirulent CIAV strains reported previously. This relatively low mortality does not deny the intrinsic high pathogenicity of the isolate. Multiple experimental conditions collectively alter the final pathogenic phenotype of CIAV infection, including the intramuscular inoculation route, uniform genetic background of SPF chickens, and the use of 1-day-old chicks as infection hosts. These influencing factors may alleviate the lethal outcome of infection and partially explain the low mortality observed in our animal challenge test. Notably, unlike certain prevalent Guangxi strains, the VP1 hypervariable region of CIAV-GDHY230813 exhibited complete sequence conservation, and key residues associated with virulence remained unchanged. This high degree of conservation may reflect strong functional constraints and could partly explain the pronounced pathogenicity observed in experimental infections. Nevertheless, several amino acid substitutions and a single deletion were identified outside the hypervariable region, indicating that mutations beyond traditionally recognised variable domains may also contribute to viral fitness, replication dynamics, and pathogenic potential, meriting further structural and functional investigation.

The VP2 and VP3 proteins are relatively conserved, assisting in the correct folding of VP1 during viral particle assembly and playing an auxiliary role in inducing neutralizing antibody production (2). VP2 functions as a dual-specificity protein phosphatase essential for proper VP1 folding and virion assembly, while VP3, also known as apoptin, induces apoptosis in host immune cells, particularly thymocytes. Although several amino acid substitutions, insertions, and a deletion were observed in VP2, no alterations were detected within its highly conserved catalytic motif, suggesting that its phosphatase activity and functional integrity are likely preserved (26). In contrast, the VP3 sequence of CIAV-GDHY230813 showed complete conservation, implying that the virus retains its capacity to induce apoptosis in lymphoid and haematopoietic tissues, thereby contributing to immunosuppression and anaemia.

This study systematically evaluated the pathogenicity of the CIAV-GDHY230813 strain isolated from southern China. The results demonstrated that the body weight of chicks in the challenged group was significantly lower than that of the control group from 3 dpi, indicating a pronounced inhibitory effect on growth performance. Furthermore, the mortality rate in the challenged group reached approximately 7%, with deaths occurring predominantly within the first 5 dpi, a pattern consistent with the infection characteristics of highly virulent CIAV strains reported previously. Infected birds also exhibited conspicuous point-like and patchy haemorrhages in the pectoral and leg muscles, as well as in subcutaneous tissues, reflecting the severe pathological damage induced by CIAV infection (27), which may contribute to the development of anaemia through the destruction of erythrocytes and myeloid-derived specialized progenitor cells (28). No dermatitis or blue wing syndrome was observed following challenge with CIAV-GDHY230813; however, severe thymic and bursal atrophy, splenomegaly, and a marked reduction in haematocrit levels were evident, indicating profound anaemia, whether there is a persistent anemia status requires further long-term monitoring.

CIAV can disseminate within chicken flocks through both horizontal and vertical transmission, with infection of the gastrointestinal tract generally regarded as the primary route of horizontal spread (29). Following challenge with CIAV-GDHY230813, the detection rate in anal swabs reached 100% as early as 14 days of age, and both anal and pharyngeal swabs consistently exhibited a 100% positivity rate at 21 days of age, indicating a high capacity for horizontal transmission. Accordingly, cloacal swabs represent the preferred sample type for the clinical detection and epidemiological surveillance of CIAV. These findings further suggest that, in the absence of specific antiviral agents and highly effective vaccines, CIAV is exceedingly difficult to eradicate once introduced into a poultry flock.

Analysis of viral tissue distribution and load further elucidated the tissue tropism of CIAV-GDHY230813. During the early stage of infection, viral loads in the spleen and bursa of Fabricius were significantly higher than those observed in the control group. As the duration of infection increased, viral loads in the blood, liver, and multiple immune organs continued to rise, indicating that CIAV-GDHY230813 is capable of sustained and efficient replication *in vivo*. These findings are consistent with previous reports demonstrating that CIAV primarily targets lymphoid tissues, particularly thymic lymphocytes, and that the virus can persist in the host for prolonged periods, rendering it difficult to achieve complete neutralization by host antibodies (30–32). Following CIAV infection, the virus can be released from the thymus into the systemic circulation, subsequently disseminating and replicating in multiple tissues and organs throughout the host, which may account for its extensive tissue distribution.

VP3 induces apoptosis in thymic CD4^+^/CD8^+^ immature T precursors, depleting peripheral helper T cells and abolishing costimulatory signals and IL-4/IL-6 critical for B-cell activation. This disrupts germinal center reactions, blocks somatic mutation and IgM to IgY switching, causing persistent dual immunosuppression, poor vaccine efficacy, and frequent secondary infections in chicks (28, 31). The observed atrophy of the thymus and bursa of Fabricius indicates damage to central immune organs, suggesting profound suppression of humoral immunity. Serum antibody titres against NDV in the CIAV-GDHY230813-infected group were significantly lower than those in the control group at both 14 and 21 days of age, indicating that this strain effectively suppresses the humoral immunity elicited by NDV vaccination. These findings are consistent with previous reports demonstrating that both single CIAV infection and co-infection with ALV-J significantly inhibit antibody production following vaccination against H9N2 and FAdV-. By suppressing host immune function, CIAV compromises the efficacy of conventional vaccines in poultry flocks, thereby rendering chickens more susceptible to secondary infections. Consequently, stringent screening for CIAV contamination is of critical importance in both vaccine production and clinical application.

## 5 Conclusion

In conclusion, this study successfully isolated a highly pathogenic CIAV strain, designated CIAV-GDHY230813, from 56-day-old Mahuang chickens in Heyuan, Guangdong Province, China. Phylogenetic analysis classified this isolate within I-a, with a complete genome length of 2,298 bp and a glutamine (Q) residue at position 394 of VP1, a molecular marker associated with high virulence. Experimental infection demonstrated that CIAV-GDHY230813 induced severe anaemia, marked immune organ atrophy, growth retardation, and approximately 6.7% mortality in SPF chicks. Furthermore, the virus exhibited sustained replication in the bloodstream and major immune organs, significantly impaired the protective efficacy of Newcastle disease vaccination, and displayed efficient horizontal transmission. Collectively, these findings expand the genomic dataset of CIAV circulating in China and provide valuable insights into its molecular epidemiology and pathogenic mechanisms, thereby contributing to the development of more effective prevention and control strategies.

## Data Availability

The data presented in the study are deposited in the National Center for Biotechnology Information repository, accession number PX496712.1.

## Supplementary material

SUPPLEMENTARY FIGURE S1 Amino acid sequence analysis of CIAV-GDHY0813 VP1.

SUPPLEMENTARY FIGURE S2 Amino acid sequence analysis of CIAV-GDHY0813 VP2.

SUPPLEMENTARY FIGURE S3 Amino acid sequence analysis of CIAV-GDHY0813 VP3.
