# Supplementary material for: Genomic and pathogenic characterization of a highly pathogenic chicken infectious anemia virus strain in China
Source: Front Vet Sci. 2026 Jul 21;13:1893535. doi: 10.3389/fvets.2026.1893535 (PMC13435667; doi:10.3389/fvets.2026.1893535)
Supplement: Supplementary file 1 [file Table_1.docx]

**Table S1** Details of CIAV reference strains

| Strains | Gene Bank accession | year | Origin |
| --- | --- | --- | --- |
| TR20 | AB027470.1 | 1999 | Japan |
| C368 | AB046589.1 | 2001 | Japan |
| C369 | AB046590.1 | 2001 | Japan |
| SMSC-I | AF285882.1 | 2003 | Malaysia |
| 98D02152 | AF311892.2 | 2016 | USA |
| Del-Ros | AF313470.1 | 2000 | USA |
| 3-1 | AF390038.1 | 2003 | Malaysia |
| SMSC-1P60 | AF390102.1 | 2003 | Malaysia |
| BD-3 | AF395114.1 | 2004 | Bangladesh |
| LF4 | AY839944.2 | 2005 | China |
| TJBD33 | AY843527.2 | 2005 | China |
| 26P4 | D10068.1 | 2007 | The Netherlands |
| AH4 | DQ124936.1 | 2005 | China |
| SH16 | DQ141671.1 | 2005 | China |
| CIAV89-69 | JF507715.1 | 2011 | South Korea |
| GD-1-12 | JX260426.1 | 2012 | China |
| GXC060821 | JX964755.1 | 2006 | China |
| CAV-10 | KJ872513 | 2014 | Argentina |
| Strains | Gene Bank accession | year | Origin |
| SC-MZ42A | KM496307 | 2014 | China |
| GD-103 | KU050678.1 | 2016 | China |
| LY-1 | KX447636.1 | 2016 | China |
| HLJ14101 | KY486136.1 | 2014 | China |
| HLJ15170 | KY486144.1 | 2015 | China |
| JL14023 | KY486145.1 | 2014 | China |
| JS15165 | KY486152.1 | 2015 | China |
| LN15169 | KY486154.1 | 2015 | China |
| CIA-1 | L14767.1 | 1996 | USA |
| Cux-1 | M55918.1 | 2008 | Germany |
| Cuxhaven | M81223.1 | 1993 | Germany |
| CAV-GZ2-2016 | MG827099 | 2018 | EgyPt |
| 17SY0902 | MK089243 | 2019 | China |
| GX1804 | MK484615.1 | 2018 | China |
| AB1K | MT259319.1 | 2016 | Turkey |
| 1860TW | MT799769.1 | 2018 | China |
| JS2020-PFV | MW234428.1 | 2020 | China |
| GX2020-D3 | MW579761.1 | 2020 | China |
| SDSPF2020 | MW660821.1 | 2020 | China |
| Strains | Gene Bank accession | year | Origin |
| Ahhui1998 | OM799843.1 | 2023 | China |
| Pigeon-CIAV-1906 | MT536347.1 | 2019 | China |
| CQ21313 | OP038327.1 | 2021 | China |
| FJ21425 | OP038331.1 | 2021 | China |
| FJ21821 | OP038334.1 | 2021 | China |
| GX21124 | OP038345.1 | 2021 | China |
| 5-IM201910 | 0Q116656.1 | 2019 | China |
| 10-AH201911 | 0Q116658.1 | 2019 | China |
| 20-SD201911 | 00116673.1 | 2019 | China |
| 704 | U65414.1 | 1996 | Austral1a |
| Isolate 18 | KJ728827 | 2014 | China |
| CIAV-F 10 | KU845735.1 | 2016 | China |
